# The Impact of Sleep Pattern in School/Work Performance During the COVID-19 Home Quarantine in Patients With Narcolepsy

**DOI:** 10.3389/fneur.2022.849804

**Published:** 2022-07-01

**Authors:** Mengke Zhao, Baokun Zhang, Jiyou Tang, Xiao Zhang

**Affiliations:** ^1^Department of Neurology, The First Affiliated Hospital of Shandong First Medical University & Shandong Provincial Qianfoshan Hospital, Jinan, China; ^2^Department of Neurology, Shandong Provincial Qianfoshan Hospital, Cheeloo College of Medicine, Shandong University, Jinan, China

**Keywords:** narcolepsy, COVID-19, home quarantine, school/work performance, sleep behavioral therapy

## Abstract

**Objectives:**

Narcolepsy patients were observed improvements in their academic performance during the COVID-19 home quarantine. Therefore, we aim to investigate the influence of sleep behavioral changes on school/work performance in narcolepsy patients during the home quarantine.

**Methods:**

Patients admitted to Shandong Provincial Qianfoshan Hospital from Jan 1, 2017 to Jan 1, 2021 who were diagnosed with narcolepsy were studied by online questionnaires in two different periods (during and 1 year after the COVID-19 home quarantine), including five aspects: (1) changes in school/work performance (percentile ranking in class/Sheehan Disability Scale 1, SDS1); (2) daytime functions; (3) clinical symptoms; (4) psychological moods; (5) medication situations.

**Results:**

A total of 46 narcolepsy patients 34 (73.9%) narcolepsy type 1, 12 (26.1%) narcolepsy type 2 with average age of 20.76 ± 8.99 years and an equal number of age and gender matched control subjects were enrolled. During the COVID-19 home quarantine, the narcolepsy patients were found that they altered sleep patterns, including later get up time (*P* < 0.001), longer total sleep time (TST, *P* = 0.001), better sleep quality (PSQI, *P* = 0.001), and lower anxiety level (*P* = 0.005). Their school/work performance improved parallelly [with better percentile ranking (*P* = 0.001) and lower SDS1 scores (*P* = 0.002)]. The results of multiple linear stepwise regression analysis showed a linear regression relationship between TST [efficient (95%) −7.356 (−13.570 to 1.143)], SDS1 score [efficient (95%) 6.580 (2.346–10.815), *P* = 0.004] and the percentile ranking after adjusting for potential effects. Both the improvements of sleep behavior and school/work performance disappeared after the end of COVID-19 home quarantine. No similar fluctuation was found in the control group.

**Discussion:**

Changes in sleep pattern during the COVID-19 home quarantine, such as longer sleep time and later wake-up time, can reduce the degree of daytime sleepiness and increase the degree of daytime wakefulness of narcolepsy patients, which can alleviate the impact of the disease on school/work performance.

## Introduction

Narcolepsy is a rare chronic sleep disorder. It is believed to be associated with immune-mediated loss of hypocretin caused by infectious factors such as influenza and genetic factors such as HLA-DQB1 and HLA-DQA1 (1–3). Its main clinical symptoms are excessive daytime sleepiness, mood-related cataplexy, sleep paralysis and hallucinations. It also has other manifestations, such as fragmented sleep at night, cognitive impairment, the influence of study and work, emotional and psychological changes, etc. It is divided into type 1 and type 2. The specific biomarker of narcolepsy type 1 is the loss of hypocretin, in addition, 98% of narcolepsy type 1 were HLA-DQB1^*^0602 positive. However, the concentration of hypocretin in CSF of narcolepsy type 2 is not low, and the positive rate of HLA-DQB1^*^0602 in the blood is significantly lower than that of narcolepsy type 1 (4, 5). A meta-analysis conducted by Zhang et al. showed that narcolepsy type 1 had more disturbed nighttime sleep than narcolepsy type 2. At the same time, objective indicators such as the decreased rapid eye movement sleep latency (REML) and the increased narcolepsy type 1 were significantly correlated with the decreased level of hypocretin. They believed that this supported that hypocretin dysfunction in narcolepsy patients would cause their night sleep disorder to a certain extent (6). Andlauer et al. found that some narcolepsy patients with low hypocretin and no cataplexy will have cataplexy with the course of the disease. In the CSF of some narcolepsy patients without cataplexy, low or uncertain levels of hypocretin may be found early. These may also partly explain the correlation between NT1 and NT2 (7, 8). Treatment for narcolepsy can be broken down into two categories: pharmaceutical therapy to address syndrome and non-pharmaceutical therapy to improve the sleep quality mainly by lifestyle modification. However, due to factors such as a long treatment cycle, high cost, and inconvenience in purchasing medications, it was difficult for patients to adhere to pharmaceutical therapy. The importance of non-pharmaceutical therapy, which was comprised by cognitive behavioral therapy (CBT-H), daytime sleepiness therapy, cognitive and behavioral therapy for cataplexy attacks, and psychological counseling was highlighted (9–11). In recent years, there is no difference between two types of narcolepsy patients in non-pharmaceutical treatment (12). In addition, some articles related to the effects of the COVID-19 on narcolepsy patients had been published at present. Among them, two articles believed that NT1 patients' lives are more affected and prone to nocturnal sleep disorder fragmentation. Therefore, they only studied the sleep patterns of NT1 patients during the quarantine. The other two articles did not divide narcolepsy patients into two types but studied them as a whole (13–16).

In order to prevent the COVID-19 epidemic, a national lockdown was started since January 24, 2020. During this period, all the schools and most of the factories in China were closed. Online classes and telecommuting were generally adopted to refrain from going outdoors. All participants in our study strictly abided by the stay-at-home policy, and all their daily activities, including study and physical exercise, were carried out indoors, which changed their sleep behaviors indirectly (17). This condition went on for about 5 months. Most of the patients, both the students and workers, made great progress in their school/work performance. However, they returned to their usual level after the end of the lockdown. Meanwhile, no similar fluctuation was found in the control group. Therefore whether such differences were related to changes in sleep pattern aroused our attention.

It was reported that patients could stay awake for longer periods of time and better reaction times when allowed to have long naps and night sleep (18). In order to describe the manifestation change of the narcolepsy patients during and 1 year after the COVID-19 home quarantine, and to analyze the possible role of sleep pattern during the home quarantine on their school/work performance, we collected and analyzed percentile ranking, work performance, bed time, get up time, narcolepsy symptoms, the degree of daytime sleepiness, and psychological moods.

## Materials and Methods

### Narcolepsy Patients and Healthy Controls

#### Inclusion Criteria

Patients admitted to Shandong Provincial Qianfoshan Hospital from Jan 1, 2017 to Jan 1, 2021 with a diagnosis of narcolepsy, according to the ICSD-3, agreed to the follow-up survey. Healthy people with matched age, sex and education level were taken as controls. All participants basically obeyed the stay-at-home policy and worked stably during the epidemic from Jan to June, 2020. All participants were informed of the purpose of the investigation and accepted the results of the study.

#### Exclusion Criteria

(1) With other diseases which cause sleepiness at the same time, such as sleep apnea respiratory syndrome, sleep deprivation syndrome and acute cerebral infarction, according to the ICSD-3; (2) Discharged patients who were unable to be contacted; (3) Patients with key data missing.

We used the percentage of the number of people who are ahead of them in class to evaluate the school performance of these students. This index was calculated *via* the class of mock exams which was information from school registers. The formula is as follows:^*^(Percentile ranking) = class rankings/the class size × 100%. The “work performance” of workers is expressed by self-evaluation of the impact of quarantine on work (SDS1).

**Figure 1:**
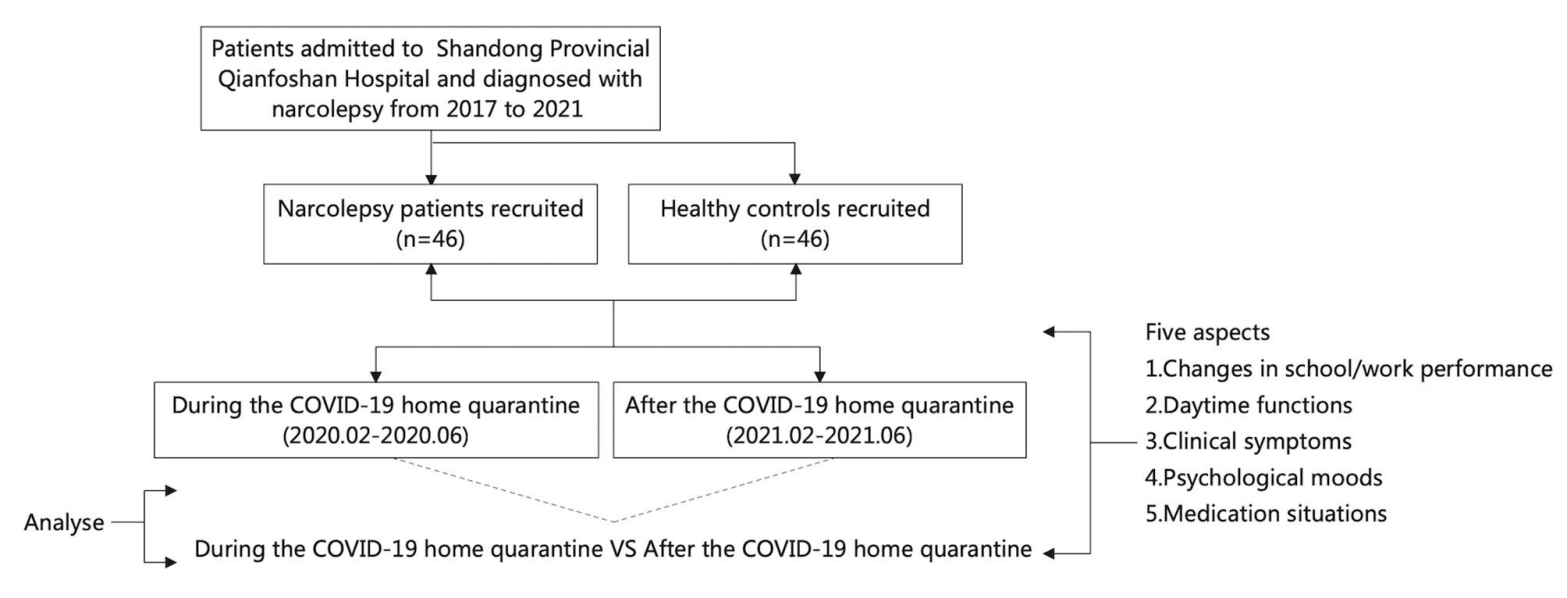
Consort diagram and participant flowchart.

All the data were obtained by telephone interviews. The flowchart is as follows (Figure 1). The questionnaire included five aspects: (1) Changes in school/work performance; (2) Daytime functions, such as the degree of daytime sleepiness (Epworth Sleepiness Scale, ESS), daytime naps and planned naps; (3) Clinical symptoms of narcolepsy (Ullanlinna Narcolepsy Scale, UNS), hallucinations, sleep paralysis; (4) Psychological moods, such as anxiety (Generalized Anxiety Disorder-7, GAD-7) and depression (Patient Health Questionnaire-9, PHQ-9); (5) Medication situations. All follow-up patients and healthy subjects were informed of the purpose of the investigation and accepted the results of the investigation. This study was approved by the Human Research Ethics committee of the Shandong Provincial Qianfoshan Hospital (Ethics approval number: YXLL-KY-2021-026).

### Statistical Analyses

IBM SPSS Statistics, version 26 (New York, United States) was used for statistical analysis. Categorical variables were expressed by percentage (%). McNemar's Chi-squared test was used to compare the same groups during and after the COVID-19 home quarantine. Pearson's chi-square test was used to compare different groups during and after the home quarantine. Continuous variables data were represented as the mean ± SD. Paired *T*-tests and the nonparametric Wilcoxon signed-rank test were used to compare the same groups during and after the COVID-19 home quarantine, independent sample *T*-tests and Mann–Whitney *U*-tests were used for comparisons between different groups during and after the home quarantine. Correlation analysis was conducted for differences in the changes in academic performance of the two groups and various sleep behavior indicators. Pearson correlation analysis was used for the data in line with a normal distribution. The Spearman rank test was used for data that did not conform to a normal distribution. Finally, linear regression analysis of the percentile ranking and various sleep behavior indicators in the narcolepsy group were performed. Univariate linear regression analysis of percentile ranking and changes in sleep patterns in the patients and healthy controls respectively. We performed further multiple linear stepwise regression analysis between the percentile ranking and SDS, TST, PSQI score, number of naps, PHQ score in patients after adjusting some potential effects, including age and BMI, grade, different medicine used. Because of the multiple comparisons, FDR (Benjamini–Hochberg procedure) was used to correct *P* values and control false positive rates.

## Results

### Baseline Characteristics and Different Sleep Patterns of Narcolepsy Patients and Controls

A total of 46 narcolepsy patients and 46 healthy controls were included in our study (see the flow chart, Figure 1). The mean age of narcolepsy patients was 20.76 ± 8.99 years, and there was no significant difference in age between the two groups (*P* = 0.461). There were 30 males (65.2%) and 16 females (34.8%), with a male to female ratio of 1.88 to 1. There was a significant difference in body mass index (BMI, = kg/m^2^) between narcolepsy patients and healthy controls (*P* < 0.001). Among patients, 35 (76.1%) had received medication, at the same time, different adverse reactions were reported. Some patients voluntarily stopped pharmaceutical treatment before or during the home quarantine, and only 10 (21.7%) insisted on the pharmaceutical treatment regularly during the COVID-19 home quarantine. Further baseline characteristics were presented in Table 1.

**Table 1:** Baseline characteristics and different sleep patterns of narcolepsy patients and controls.

**Characteristics**	**Narcopelsy (*n* = 46)**	**Controls (*n* = 46)**	***x*** **^2^/t**	**P**
	**Mean ±SD**	**Mean ±SD**		
Age, year	20.76 ± 8.99	19.89 ± 9.14	−0.737	0.461^*^
**Sex, (%)**				
Male	30 (65.2)	27 (58.7)	0.415	0.519
Female	16 (34.8)	19 (41.3)		
BMI, kg/m^2^	26.49 ± 4.66	20.45 ± 3.38	4.270	<0.001
Appetite change, (%)	15 (32.6)	NA		
Temperamental change, (%)	32 (69.6)	NA		
School/Work influence, (%)	32 (69.6)	NA		
**Current status, (%)**				
Primary school	3 (6.5)	3 (6.5)	6.594	0.159
Junior middle school	8 (17.4)	12 (26.1)		
Senior high school	8 (17.4)	13 (28.3)		
College	16 (34.8)	15 (32.6)		
Work	11 (23.9)	3 (6.5)		
Medication therapy, (%)	35 (76.1)	NA		
Take medicine regularly during the COVID-19 home quarantine, (%)	10 (21.7)	NA		
**Type**				
NT1	34 (73.9)	NA		
NT2	12 (26.1)	NA		
	**During the COVID-19 home quarantine**		***x*** **^2^/** ***Z***	***P*** **-value**
Weight gain, (%)	22 (47.8)	13 (28.3)	3.735	0.053
Prolonged sleep at night, (%)	24 (52.2)	23 (50)	0.043	0.835
Percentile ranking	42.25% ± 28.02%^a^	36.26% ± 22.96%^b^	−0.904	0.366
SDS1 score	2.54 ± 2.26	2.43 ± 2.29	−0.186	0.852
ESS score	12.76 ± 5.62	5.43 ± 5.13	−5.610	<0.001
**Night sleep structure**				
Bed time, hh:mm	22:31 ± 1:00	22:51 ± 0:54	−1.756	0.079
Sleep latency, min	11.28 ± 9.68	18.76 ± 11.31	−3.665	<0.001
Get up time, hh:mm	7:22 ± 1:17	7:20 ± 0:50	−0.276	0.783
TST, h	7.84 ± 1.68	7.73 ± 1.10	−0.723	0.469
PSQI score	3.87 ± 2.43	3.78 ± 2.04	−0.399	0.690
GAD-7 score	4.09 ± 4.32	2.63 ± 4.23	−2.300	0.021
PHQ-9 score	6.96 ± 5.18	2.96 ± 4.63	−4.182	<0.001

* *Mann–Whitney U-tests, t-test for ranks; x^2^, pearsons chi-square test; t, t-test; Z, Mann–Whitney U-test*.

a *35 peoples*.

b *43 peoples*.

*BMI, body mass index; n, number; SD, standard deviation; NT1, narcolepsy type 1; NT2, narcolepsy type 2; SDS1, Sheehan Disability Scale; ESS, Epworth Sleepiness Score; TST, Total sleep time; PSQI, Pittsburgh Sleep Quality Index; GAD-7, Generalized Anxiety Disorder-7; PHQ-9, Patient Health Questionnaire-9*.

Compared to healthy controls, narcolepsy patients had more severe EDS (ESS score, 12.76 ± 5.62 vs. 5.43 ± 5.13, *P* < 0.001), faster sleep latency (11.28 ± 9.68 vs. 18.76 ± 11.31, *P* < 0.001), more anxiety (GAD-7 score, 4.09 ± 4.32 vs. 2.63 ± 4.23, *P* = 0.021), more depression (PHQ-9 score, 6.96 ± 5.18 vs. 2.96 ± 4.63, *P* < 0.001), and relatively lower academic performance (the difference was not significant).

### The Sleep Pattern and School Performance Were Affected by the COVID-19 Home Quarantine in Narcolepsy Patients

During the home quarantine, students had the later get up time (7:27 ± 1:16 vs. 6:28 ± 0:52, *P* < 0.001), longer TST (8.19 ± 1.37 vs. 7.37 ± 1.20, *P* = 0.001), longer sleep latency (12.37 ± 10.29 vs. 9.91 ± 6.37, *P* = 0.032) and longer nap duration (19.86 ± 9.66 vs. 15.14 ± 10.25, *P* = 0.007), better sleep quality (PSQI score, 3.46 ± 2.01 vs. 4.60 ± 2.37, *P* = 0.001), and lower anxiety level (GAD-7 score, 3.71 ± 4.51 vs. 5.57 ± 5.15, *P* = 0.004). Meanwhile, an improved class ranking (42.25% ± 28.74% vs. 52.91% ± 26.84%, *P* = 0.001) and a lower effect of illness on self-rated school/work (SDS1 score, 2.17 ± 2.01 vs. 3.34 ± 2.81, *P* = 0.002) were observed in students. In addition, the number of daytime naps increased, EDS decreased (ESS score), and clinical symptoms of narcolepsy decreased (UNS score). Meanwhile, the TST of workers was also prolonged (6.73 ± 2.14 vs. 6.05 ± 1.65, *P* = 0.026), and the sleep quality was better (PSQI score, 5.18 ± 3.22 vs. 6.73 ± 2.14, *P* = 0.006). However, there was no significant difference in daytime naps, EDS, UNS, anxiety and depression in workers. Details was presented in Table 2.

**Table 2:** The sleep pattern and school performance were affected by the COVID-19 home quarantine in narcolepsy patients.

**Characteristics**	**During the COVID-19 home quarantine**		**After the COVID-19 home quarantine**		**During the home quarantine vs. after the home quarantine**					
	**Students (*n* = 35)**	**Workers (*n* = 11)**	**Students (*n* = 35)**	**Workers (*n* = 11)**	**Students (*****n*** **= 35)**		**Workers (*****n*** **= 11)**		**Overall (*****n*** **= 46)**	
	**Mean ±SD**	**Mean ±SD**	**Mean ±SD**	**Mean ±SD**	***x*** **^2^/t**	***P*** **-value**	***x*** **^2^/t**	***P*** **-value**	***x*** **^2^/t**	***P*** **-value**
Weight gain, (%)	18 (51.4)	4 (36.4)	11 (31.4)	4 (36.3)	2.769	0.096		1	4.083	0.043
Prolonged sleep, (%)	21 (60)	3 (27.3)	NA	NA	NA	NA	NA	NA	NA	NA
Medicine, (%)	9 (25.71)	1 (9.1)	NA	NA	NA	NA	NA	NA	NA	NA
Percentile ranking	42.25% ± 28.74%	NA	52.91% ± 26.84%	NA	−3.283	0.001^*^	NA	NA	NA	NA
SDS1 score	2.17 ± 2.01	3.73 ± 2.69	3.34 ± 2.81	5.00 ± 2.97	−3.159	0.002^*^	−1.472	0.172	−3.392	0.001^*^
**Clinical symptoms**										
Sleepiness, (%)	32 (91.4)	9 (81.8)	31 (88.6)	9 (81.8)		1		1		1
Emotional behavior, (%)	23 (65.7)	8 (72.7)	24 (68.6)	9 (81.8)		1		1		1
Hallucination, (%)	10 (28.6)	8 (72.7)	8 (22.9)	6 (54.5)	0.250	0.617	0.5	0.5	1.5	0.221
Sleep paralysis, (%)	19 (54.3)	5 (45.5)	17 (48.6)	4 (36.3)	0.167	0.683		1	0.571	0.450
**Degree of sleepiness**										
Number of naps	3.03 ± 1.95	2.64 ± 0.92	2.83 ± 1.96	2.45 ± 0.93	−1.426	0.154	−1.414	0.157^*^	−1.734	0.083^*^
Duration of each nap, min	19.86 ± 9.66	16.36 ± 9.77	15.14 ± 10.25	18.18 ± 15.01	−2.702	0.007	−0.422	0.673^*^	−2.213	0.027^*^
ESS score	12.74 ± 5.64	12.82 ± 5.81	13.03 ± 6.20	13.09 ± 5.79	−0.329	0.744	−0.255	0.804	−0.284	0.776
**Emotional behaviors**										
Inability, (%)	22 (62.9)	8 (72.7)	23 (65.7)	8 (72.7)		1		1		1
Open mouth, (%)	13 (37.1)	4 (36.3)	14 (40)	3 (27.3)		1		1		1
Nod, (%)	17 (48.6)	5 (45.5)	16 (45.7)	5 (45.5)		1		1		1
Tumble, (%)	9 (25.7)	2 (18.2)	8 (22.9)	3 (27.3)		1	0.250	0.617		1
UNS score	15.14 ± 6.88	15.27 ± 5.53	15.17 ± 6.90	17.09 ± 8.51	−0.038	0.970	−1.023	0.306^*^	−0.672	0.502
**Night sleep structure**										
Bed time, hh:mm	22:28 ± 0:54	22:43 ± 1:19	22:30 ± 0:42	22:21 ± 0:38	−0.123	0.902^*^	−1.289	0.197^*^	−0.450	0.653^*^
Sleep latency, min	12.37 ± 10.29	7.82 ± 6.65	9.91 ± 6.37	10.73 ± 14.01	−2.147	0.032^*^	−0.921	0.357^*^	−1.531	0.126^*^
Get up time, hh:mm	7:27 ± 1:16	7:06 ± 1:21	6:28 ± 0:52	6:30 ± 0:43	−3.786	<0.001^*^	−1.590	0.112^*^	−4.100	<0.001^*^
TST, h	8.19 ± 1.37	6.73 ± 2.14	7.37 ± 1.20	6.05 ± 1.65	−3.430	0.001^*^	−2.226	0.026^*^	−3.989	<0.001^*^
PSQI score	3.46 ± 2.01	5.18 ± 3.22	4.60 ± 2.37	6.55 ± 3.70	−3.330	0.001^*^	−2.754	0.006^*^	−4.120	<0.001^*^
GAD-7 score	3.71 ± 4.51	5.27 ± 3.61	5.57 ± 5.15	5.73 ± 4.92	−2.880	0.004	−0.255	0.598	−2.788	0.005^*^
PHQ-9 score	6.91 ± 5.69	7.09 ± 3.24	7.09 ± 5.82	7.91 ± 2.43	−0.303	0.764	−0.417	0.677^*^	−1.276	0.202^*^

* *Wilcoxon signed-rank test, Paired-samples t-test for ranks; x^2^, McNemar's Chi-squared test; t, Paired-samples t-test*.

*SDS1, Sheehan Disability Scale; ESS, Epworth Sleepiness Score; UNS, Ullanlinna Narcolepsy Scale; TST, Total sleep time; PSQI, Pittsburgh Sleep Quality Index; GAD-7, Generalized Anxiety Disorder-7; PHQ-9, Patient Health Questionnaire-9; n, number; SD, standard deviation*.

### The Sleep Pattern Rather Than the School Performance Was Affected by the COVID-19 Home Quarantine in Healthy Controls

As shown in Table 3, the healthy controls were more affected by the home quarantine at school/work performance (SDS1 score, 2.43 ± 2.29 vs. 1.54 ± 1.52, *P* < 0.001), experienced more severe EDS (ESS score, 5.43 ± 5.13 vs. 4.52 ± 5.37, *P* = 0.003), delayed get up time (7:20 ± 0:50 vs. 6:56 ± 0:49, *P* = 0.001), longer TST (7.73 ± 1.10 vs. 7.26 ± 1.06, *P* = 0.003), and worse sleep quality (PSQI score, 7.73 ± 1.10 vs. 7.26 ± 1.06, *P* = 0.003). At the same time, we observed that during the pandemic period, their academic performance was lower, latency for falling asleep was prolonged, while anxiety and depression were aggravated, though there were no significant differences in these aspects.

**Table 3:** The sleep pattern rather than the school performance was affected by the COVID-19 home quarantine in healthy comtrols.

	**During the COVID-19 home quarantine**	**After the COVID-19 home quarantine**	***x*** **^2^/Z**	***P*** **-value**
	**Healthy controls (*n* = 46)**	**Healthy controls (*n* = 46)**		
	**Mean ±SD**	**Mean ±SD**		
Weight gain, (%)	13 (28.3)	9 (19.6)	0.900	0.343
Percentile ranking^a^	36.26% ± 22.96%	34.66% ± 19.62%	−0.659	0.510
SDS1 score	2.43 ± 2.29	1.54 ± 1.52	−3.489	<0.001
ESS score	5.43 ± 5.13	4.52 ± 5.37	−2.941	0.003
**Night sleep structure**				
Bed time, hh:mm	22:51 ± 0:54	22:50 ± 1:02	−0.683	0.495
Sleep latency, min	18.76 ± 11.31	18.30 ± 10.68	−0.182	0.855
Get up time, hh:mm	7:20 ± 0:50	6:56 ± 0:49	−3.374	0.001
TST, h	7.73 ± 1.10	7.26 ± 1.06	−2.988	0.003
PSQI score	3.78 ± 2.04	2.72 ± 2.04	−2.950	0.003
GAD-7 score	2.63 ± 4.23	2.02 ± 3.68	−1.821	0.069
PHQ-9 score	2.96 ± 4.63	2.78 ± 4.39	−0.809	0.418

*x^2^, McNemar's Chi-squared test; Z, Wilcoxon signed-rank test*.

a *35 peoples*.

*SDS1, Sheehan Disability Scale; ESS, Epworth Sleepiness Score; TST, Total sleep time; PSQI, Pittsburgh Sleep Quality Index; GAD-7, Generalized Anxiety Disorder-7; PHQ-9, Patient Health Questionnaire-9; n, number; SD, standard deviation*.

### In Narcolepsy Patients, Changes in Sleep Patterns and Improved School Performance Showed a More Obvious Correlation

**Figure 2:**
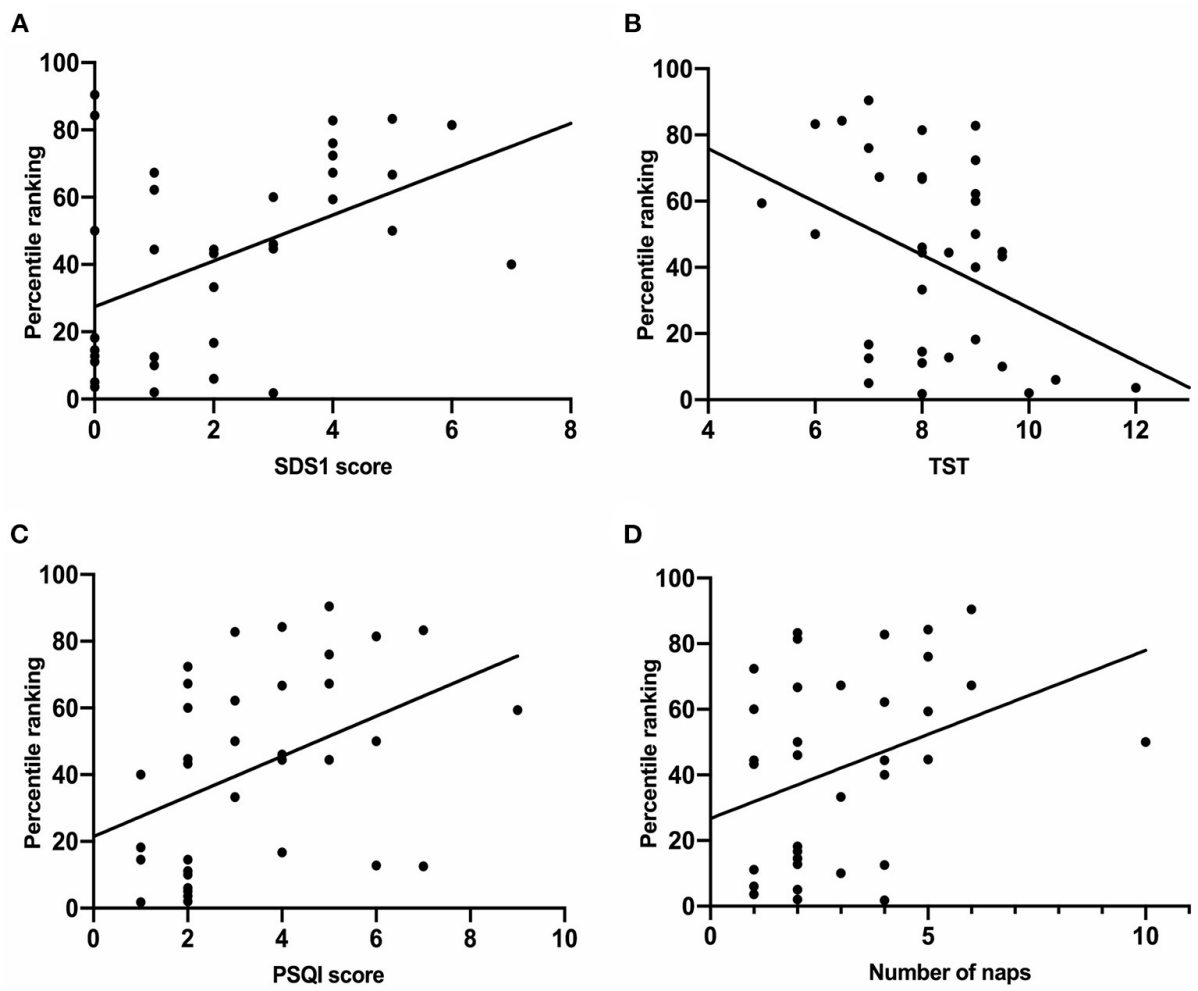
Linear regression analysis of the percentile ranking and various sleep behavior indices in narcolepsy group. **(A)** The percentile ranking of students is significantly positively correlated with SDS1 (*R* = 6.811; 95%CI: 2.351, 11.271; *P* = 0.04). **(B)** The percentile ranking of students is significantly positively correlated with PSQI score (*R* = 6.012; 95%CI: 1.405, 10.620; *P* = 0.012). **(C)** The percentile ranking of students is significantly positively correlated with numbers of naps (*R* = 5.122; 95%CI: 0.221, 10.022; *P* = 0.041). **(D)** The percentile ranking of students is significantly positively correlated with TST (*R* = −8.019; 95%CI: −14.865, −1.173; *P* = 0.023). SDS1, Sheehan Disability Scale; PSQI, Pittsburgh Sleep Quality Index; ESS, Epworth Sleepiness Score; TST, Total sleep time.

As shown in Table 4, the percentile ranking of narcolepsy patients during the home quarantine is correlated with the degree of SDS1 (*r* = 0.422, *P* = 0.012), TST (*r* = −0.129, *P* = 0.034), sleep quality (PSQI score, *r* = 0.488, *P* = 0.003), number of daily naps (*r* = 0.361, *P* = 0.033), and degree of depression (PHQ-9 score, *r* = 0.390, *P* = 0.020). After the home quarantine, the percentile ranking of narcolepsy patients was correlated with the degree of SDS1 (*r* = 0.705, *P* < 0.001), bedtime (*r* = 0.399, *P* = 0.018), and number of daily naps (*r* = 0.361, *P* = 0.001). There was no significant correlation between the percentile ranking of healthy controls during the home quarantine and any sleep pattern indices. After the home quarantine, it was correlated with sleep latency (*r* = 0.306, *P* = 0.046) and TST (*r* = −0.324, *P* = 0.034). As show in Figure 2, during the home quarantine, the increased percentile ranking of narcolepsy patients is related to the decreased degree of learning affected by pandemic (SDS1, *r* = 6.811; 95%CI: 2.351, 11.271; *P* = 0.04), better sleep quality (PSQI score, *r* = 6.012; 95%CI: 1.405, 10.620; *P* = 0.012), decreased number of naps (*r* = 5.122; 95%CI: 0.221, 10.022; *P* = 0.041) and prolonged TST (*r* = −8.019; 95%CI: −14.865, −1.173; *P* = 0.023).

**Table 4:** In narcolepsy patients, changes in sleep patterns and improved school performance showed a more obvious correlation.

	**The percentile ranking of narcolepsy patients (*****n*** **= 35)**						**The percentile ranking of healthy controls (*****n*** **= 43)**					
	**During the COVID-19 home quarantine**			**After the COVID-19 home quarantine**			**During the COVID-19 home quarantine**			**After the COVID-19 home quarantine**		
	***r***	***P*** **-value**	***P*** **(FDR)**	***r***	***P*** **-value**	***P*** **(FDR)**	***r***	***P*** **-value**	***P*** **(FDR)**	***r***	***P*** **-value**	***P*** **(FDR)**
SDS1	0.422	0.012	0.072	0.705	<0.001	<0.001	0.260	0.092	0.414	0.238	0.124	0.223
Bed time	−0.073	0.679	0.679	0.399	0.018	0.072	0.273	0.076	0.684	0.293	0.060	0.135
Sleep latency	−0.129	0.459	0.501	−0.032	0.857	0.857	0.227	0.143	0.322	0.306	0.046	0.207
Get up time	−0.269	0.119	0.238	−0.185	0.289	0.385	0.159	0.307	0.461	0.247	0.124	0.186
TST	−0.359	0.034	0.082	−0.338	0.047	0.141	−0.255	0.099	0.297	−0.324	0.034	0.306
PSQI score	0.488	0.003	0.036	0.146	0.403	0.484	0.144	0.356	0.401	0.296	0.054	0.162
Number of naps	0.361	0.033	0.099	0.528	0.001	0.006	NA	NA	NA	NA	NA	NA
Duration of each nap	−0.265	0.125	0.214	0.062	0.722	0.788	NA	NA	NA	NA	NA	NA
ESS score	0.163	0.348	0.418	0.288	0.093	0.159	0.154	0.326	0.419	0.072	0.653	0.735
UNS score	0.223	0.197	0.263	0.192	0.268	0.402	NA	NA	NA	NA	NA	NA
GAD score	0.262	0.129	0.194	0.297	0.084	0.168	0.215	0.167	0.301	−0.047	0.769	0.769
PHQ score	0.390	0.020	0.08	0.318	0.063	0.151	0.096	0.539	0.539	−0.190	0.227	0.292

*P(FDR), Benjamini–Hochberg procedure; SDS1, Sheehan Disability Scale; TST, Total sleep time; PSQI, Pittsburgh Sleep Quality Index; ESS, Epworth Sleepiness Score; UNS, Ullanlinna Narcolepsy Scale; GAD-7, Generalized Anxiety Disorder-7; PHQ-9, Patient Health Questionnaire-9*.

### Univariate and Multiple Linear Stepwise Regression Analysis Were Used to Determine the Percentile Ranking and Changes in Sleep Patterns in the Patients and Healthy Controls Respectively

There was a univariate linear regression relationship between tthe percentile ranking and SDS [6.811 (2.351–11.271), *P* = 0.004], TST [−8.019 (−14.865 to −1.173), *P* = 0.023], PSQI score [6.012 (1.405–10.620), *P* = 0.012], number of naps [5.122 (0.221–10.022), *P* = 0.041], PHQ score [2.019 (0.379–3.658), *P* = 0.017] in patients. After FDR corrected *P* values and controlled the false positive rate, only SDS1 had a linear regression relationship with the percentile ranking. We performed further multiple linear stepwise regression analysis, there was a linear regression relationship between the percentile ranking and TST [efficient (95%) −7.356 (−13.570 to 1.143), SDS1 score (95%) 6.580 (2.346–10.815), *P* = 0.004] after categorization for potential effects. As shown in Table 5. However, there was no significant linear regression relationship between the percentile ranking and changes in sleep patterns in the healthy controls.

**Table 5:** Univariate and multiple linear stepwise regression analysis were used to determine the percentile ranking and changes in sleep patterns in the patients and healthy controls respectively.

**During the COVID-19 home quarantine**										
**The percentile ranking of narcolepsy patients (*****n*** **= 35)**										
	**Univariate analysis**					**Multivariate analysis**				
	**Cofficient (95% CI)**	**Adjusted *R*^2^**	***df***	***F*** **-value**	***P*** **/** ***P*** **(FDR)**	**Cofficient (95% CI)**	**Adjusted *R*^2^**	***df***	***F*** **-value**	***P*** **-value**
SDS1	6.811 (2.351 to 11.271)	0.203	33	9.652	0.004/0.048	6.580 (2.346 to 10.815)	0.186	29	2.552	0.004
Bed time	−0.001 (−0.004 to 0.002)	−0.021	33	0.285	0.597/0.597					
Sleep latency	−0.286 (−1.269 to 0.698)	−0.019	33	0.350	0.558/0.609					
Get up time	−0.002 (−0.004 to 0.001)	0.030	33	2.047	0.162/0.243					
TST	−8.019 (−14.865 to −1.173)	0.121	33	5.679	0.023/0.069	−7.356 (−13.570 to −1.143)	0.303	28	3.465	0.022
PSQI score	6.012 (1.405 to 10.620)	0.151	33	7.048	0.012/0.072					
Number of naps	5.122 (0.221 to 10.022)	0.094	33	4.522	0.041/0.098					
Duration of each nap	−0.817 (−1.829 to 0.196)	0.047	33	2.691	0.110/0.189					
ESS score	1.177 (−0.578 to 2.931)	0.025	33	1.862	0.182/0.243					
UNS score	0.925 (−0.518 to 2.368)	0.020	33	1.700	0.201/0.241					
GAD score	1.954 (−0.194 to 4.103)	0.067	33	3.425	0.073/0.146					
PHQ score	2.019 (0.379 to 3.658)	0.134	33	6.274	0.017/0.068					
**The percentile ranking of healthy controls (*****n*** **= 43)**										
	**Univariate analysis**					**Multivariate analysis**				
	**Cofficient(95% CI)**	**Adjusted *R*^2^**	***df***	***F***	***P*** **/** ***P*** **(FDR)**	**Cofficient (95% CI)**	***P*** **-value**			
SDS1	1.886 (−1.249 to 5.021)	0.011	41	1.476	0.231/0.693					
Bed time	0.002 (0 to 0.004)	0.044	41	2.930	0.094/0.423					
Sleep latency	0.366 (−0.249 to 0.981)	0.010	41	1.443	0.236/0.531					
Get up time	0.001 (−0.001 to 0.004)	0.006	41	1.261	0.268/0.482					
TST	−5.958 (−12.050 to 0.133)	0.065	41	3.902	0.055/0.495					
PSQI score	1.441 (−1.990 to 4.872)	−0.007	41	0.719	0.401/0.516					
ESS score	0.615 (−0.757 to 1.988)	−0.004	41	0.820	0.371/0.557					
GAD score	0.360 (−1.304 to 2.024)	−0.020	41	0.191	0.664/0.747					
PHQ score	0.179 (−1.354 to 1.712)	−0.023	41	0.056	0.815/0.815					

*Model: adjusting for age and BMI, grade, different medicine used*.

*P(FDR), Benjamini–Hochberg procedure; CI, confidence nterval; SDS1, Sheehan Disability Scale; TST, Total sleep time; PSQI, Pittsburgh Sleep Quality Index; ESS, Epworth Sleepiness Score; UNS, Ullanlinna Narcolepsy Scale; GAD-7, Generalized Anxiety Disorder-7; PHQ-9, Patient Health Questionnaire-9*.

## Discussion

As a lifelong disease, narcolepsy was reported to destroy the stability of the cortex and induced a decline in attention, reaction, executive ability and cognitive function (19–22). As a significant number of patients have the disease onset in adolescents, which is considered as the golden period for learning in one's life (23, 24), it was important to find the potential influence on school/work performance. The COVID-19 lockdown in 2020 was proved to have a significant influence on lifestyle and sleep behaviors (13–16). However, previous studies mainly focused on changes of sleep behavior during the COVID-19 lockdown, there were limited data on the influence of sleep behavior on school/work performance. In this retrospective single center study, we presented the baseline characteristics, percentile ranking, work performance, sleep patterns, narcolepsy symptoms, the degree of daytime sleepiness, psychological moods and found that there was a correlation between the degree of changes in school/work performance and various sleep behaviors. Delayed waking time in the morning, prolonged TST at night, and lowered depression degree could help EDS of narcolepsy patients be alleviated, improve their daytime arousal level, and further improve their school/work performance.

Early observations consistently indicated that the fragmentation of nocturnal sleep in patients with narcolepsy affects the quality and brings pain to patients (25). Inocente et al. (26) argued that the clinical symptoms of narcolepsy caused them to experience physical fatigue, decreased school performance time spent on home life, social activities, and school performances. Jennum et al. (27) concluded that the working ability of adults with narcolepsy was affected to some extent; thus, their employment rate and income level were lower than those of the control group. To better reduce the negative impact of the disease on patients with narcolepsy, the UK consensus emphasizes the importance of non-drug treatment (28). Recent cohort studies in narcolepsy showed that patients' sleep patterns during the COVID-19 pandemic changed. For example, Rodrigues Aguilar et al. found that the sleep-wake time of narcolepsy patients was not fixed (15). Wu et al. (16) found that delaying the get up time and increasing TST at night could effectively alleviate patients' daytime sleepiness. The same trend was found in our study. However, we did not find any difference in bed-time. That maybe because in our study, a large proportion of the participants were students, and most of them followed a strict routine. A fixed bedtime was usually part of the routine.

During the home quarantine, compared with the control group, the sleep latency of patients was significantly shorter. At the same time, their degree of daytime sleepiness was heavier and more prone to emotional changes, such as depression. The apparent reduction of sleep latency and the disorder of sleep rhythm are the characteristics of narcolepsy patients. To further study their sleep patterns during quarantine, we studied the different sleep patterns of narcolepsy patients and healthy controls during and after the COVID-19 home quarantine. We divided the patient group into students who took online courses at home and people who worked from home. During quarantine, the sleep latency of narcolepsy students increased by 2.5 ± 3.9 min, the time to get up was delayed by nearly 1 h, the TST increased by 1.5 h, and their sleep quality improved. However, narcolepsy workers only showed an increase in TST (0.7 ± 0.5 h) and an improvement in sleep quality during the home quarantine. Consistent with Wu's study (16), no significant change was found at bedtime, although the get up time and the TST changed. When the get up time and TST of narcolepsy patients and healthy controls increased, the sleep quality had the opposite results. For example, the time to get up was delayed by 24 ± 1 min, the TST increased by 0.5 ± 0.04 h, but their sleep quality deteriorated. We considered that the anxiety degree of healthy controls increased during the home quarantine, which led to the extension of sleep latency and the decline of sleep quality. However, the sleep latency of narcolepsy patients was shorter than that of healthy controls, and the degree of anxiety decreased during the home quarantine. These were conducive to the appropriate extension of sleep latency and improved sleep quality (29, 30). The degree of these changes in healthy controls was less than that in narcolepsy patients, which suggested that the sleep pattern of narcolepsy patients was more vulnerable to behavioral changes. During the home quarantine, both groups were able to get more sleep because of the shift from in-person schooling to online education, thus saving travel time to school. In terms of symptoms of narcolepsy, Aguilar's study found that subjective sleepiness worsened, but hallucinations decreased during the home quarantine. Postiglione' et al. found no significant changes in the degree of sleepiness in patients. To increase the reliability of the study, during the quarantine period, Filardi et al. performed follow-up activities on 18 NT1 patients who regularly took sodium oxybate. Different from subjective evaluation, body motion is an objective index to evaluate sleep patterns, which is more reliable to a certain extent. Their results showed the patients delayed the bedtimes and wake-up times, prolonged the TST at night and increased the number of naps, but the duration of each nap, night sleep quality and subjective sleepiness did not change. They argued that patients took medication regularly during quarantine, so their sleep quality had not changed significantly. In our study, we observed decreasing trends in ESS and UNS scores of narcolepsy during pandemic (the differences were not significant), and a decreasing trend in anxiety level. Narcolepsy patients are prone to comorbidities of neurological diseases (31–33). We found that the narcolepsy group had higher anxiety and depression than the control group. The mood of narcolepsy patients improved during the quarantine period, which is related to the reduction of symptoms after behavioral changes and the relative rdecrease in its influence on daytime arousal and school/work performance. At the same time, the level of anxiety and depression in the healthy controls increased, which may in part result from the fact that during the pandemic period, many students did not adapt to the online learning environment and lacked the motivation for autonomous learning. Some studies have found that 36.8% (34) of students prefer “face-to-face” learning to online learning alone.

Since January 24, 2020, in order to prevent the COVID-19 epidemic, all the schools and most of the factories in China kept closed for 5 months, and all the students took online classes to refrain from going outdoors. During this period, all participants in our study strictly abided by the stay-at-home policy and all daily activities. Our advantage is to study the changes in school/work performance and sleep patterns of narcolepsy patients during the COVID-19 home quarantine. Given the non-pharmaceutical therapy of narcolepsy patients, we investigated the correlation between learning or work performance and these changes in sleep patterns. We used the percentage of the number of people who are ahead of them in class to evaluate the school performance of these students. This index was calculated *via* the class of mock exams which was information from school registers. The percentile ranking of students during the home quarantine was better than that after the quarantine, which was related to the influence of the decreased degree of illness on learning (*r* = 0.422), the increase of TST (*r* = −0.359), the improvement of sleep quality (*r* = 0.488), the decrease of daily naps number (*r* = 0.361), and the decrease of depression (*r* = 0.390). It is suggested that the “behavioral therapy” of home quarantine can promote the change of sleep pattern to some extent, thereby improving the study performance of narcolepsy patients. Contrary to narcolepsy patients, the school/work performance of healthy controls were believed to have been more affected during the home quarantine, and their grade percentage decreased, however, there was no significant correlation between the percentile ranking and various sleep pattern indices, which was considered to be related to a variety of factors, among which the increased anxiety and depression of healthy people affected academic performance, which was consistent with the literature report (35). During the home quarantine, workers had longer TST and better sleep quality; however, there was no significant change in the degree of their self-rated work affected by the illnesses. There was a univariate linear regression relationship between the percentile ranking and SDS, TST, PSQI score, number of naps, PHQ score in patients. After FDR corrected *P*-values and controlled the false positive rate, only SDS1 had a linear regression relationship with the percentile ranking. We performed further multiple linear stepwise regression analysis between the percentile ranking and SDS, TST, PSQI score, number of naps, PHQ score in patients after adjusting some potential effects, including age and BMI, grade, different medicine used. And there was a linear regression relationship between the percentile ranking and TST, SDS1 score. However, in the healthy controls, there was no significant linear regression relationship between the percentile ranking and changes in sleep patterns. Research reported (13) that patients who continued to work normally during the outbreak only showed a decrease in the quality of sleep, an increase in TST and number of daytime naps, and they also showed reduced EDS, while the unemployed had no obvious clinical changes, which was considered to be related to different working statuses. However, this study proved that improving the sleep schedule at night, rationally arranging daytime naps and improving the mood of narcolepsy patients can impact their academic performance ranking to some extent.

The major limitation of the study was related to the disease's rare nature. We enrolled totally 46 narcolepsy patients in this study, including 34 narcolepsy type 1 and 12 narcolepsy type 2. The relatively small sample made further stratified studies impossible. Among all the 46 patients, three were primary school students, eight were junior middle school students, eight were senior high school students, 16 were college students, 11 were workers; stratified study according to different ages and grades would provide better theoretical guidance in other populations. Secondly, this study is based on subjective self-report measurement and therefore, bias was inevitable. In subsequent studies, the scale would be simplified as much as possible to increase patients' understanding, and objective indicators such as sleep monitoring could be used for verification. Third, we can guide sleep behavior changes to study the behavior changes and the academic school/work performance of type 1 and type 2 under behavioral intervention.

In conclusion, a retrospective study of 46 narcolepsy patients indicated that prolonged TST in their sleep pattern was associated with improvement in school/work, life schedule, and clinical symptoms. Non-pharmaceutical therapy could effectively improve daytime wakefulness as well as school/work performance, reduce daytime sleepiness and the adverse effects on daily life. To some extent, it was more important in the treatment of some patients due to poor access to medication for economic reasons. A prospective multi-centered study with larger scale is needed to confirm these findings.

## Data Availability Statement

The original contributions presented in the study are included in the article/Supplementary Material, further inquiries can be directed to the corresponding author.

## Ethics Statement

The studies involving human participants were reviewed and approved by the Human Research Ethics Committee of the Shandong Provincial Qianfoshan Hospital. Written informed consent to participate in this study was provided by the participants' legal guardian/next of kin.

## Author Contributions

Material preparation and data collection and analysis were performed by MZ, BZ, JT, and XZ. The first draft of the manuscript was written by MZ. All authors commented on previous versions of the manuscript. All authors contributed to the study conception and design. All authors read and approved the final manuscript.

## Funding

This work was supported by the Natural Science Foundation of Shandong Province (ZR2020MH160).

## Conflict of Interest

The authors declare that the research was conducted in the absence of any commercial or financial relationships that could be construed as a potential conflict of interest.

## Publisher's Note

All claims expressed in this article are solely those of the authors and do not necessarily represent those of their affiliated organizations, or those of the publisher, the editors and the reviewers. Any product that may be evaluated in this article, or claim that may be made by its manufacturer, is not guaranteed or endorsed by the publisher.

## Abbreviations

COVID-19: Corona Virus Disease 2019
SDS1: Sheehan Disability Scale 1
TST: Total Sleep Time
PSQI: Pittsburgh Sleep Quality Index
HLA: Human Leukocyte Antigen
CBT-H: Cognitive behavioral Therapy
ICSD-3: International Classification of Sleep Disorders - Third Edition (ICSD-3)
NT1: narcolepsy type 1
NT2: narcolepsy type 2
ESS: Epworth Sleepiness Scale
UNS: Ullanlinna Narcolepsy Scale
GAD-7: Generalized Anxiety Disorder-7
PHQ-9: Patient Health Questionnaire-9.
