# Supplementary material for: The Impact of Sleep Pattern in School/Work Performance During the COVID-19 Home Quarantine in Patients With Narcolepsy
Source: Front Neurol. 2022 Jul 1;13:849804. doi: 10.3389/fneur.2022.849804 (PMC9283684; doi:10.3389/fneur.2022.849804)
Supplement: Supplementary file 1 [file Table_1.DOCX]

Supplementary Material

**Table 1.** The patient group is substantial (n=68) considering that narcolepsy is an uncommon disorder, but it is disappointing that the study sample is only 2/3 of the total number of subjects asked to participate. There are no differences (age, sex, occupation) between those who agreed to participate and those who refused. They did not agree to participate in the follow-up mainly from the following two aspects: 1. Failed to contact themselves; 2. They said there was no time to be reluctant to participate in the investigation.

| Characteristics |  | Narcolepsy patients who agreed to participate (n=46) | | Narcolepsy patients who refused to participate (n=22) | P |
| --- | --- | --- | --- | --- | --- |
|  |  | Mean ± SD | Mean ± SD | |  |
| Age, y |  | 20.76 ± 8.99 | 20.73 ± 12.04 | | 0.98 |
| Sex, (%) | Male | 30 (65.2) | 15 (68.2) | | 0.82 |
|  | Female | 16 (34.8) | 9 (40.9) | |  |
| Current status, (%) | Primary school | 3 (6.5) | 1 (4.5) | | 0.57 |
|  | Junior middle school | 8 (17.4) | 6 (27.3) | |  |
|  | Senior high school | 8 (17.4) | 6 (27.3) | |  |
|  | College | 16 (34.8) | 4 (18.2) | |  |
|  | Work | 11 (23.9) | 5 (22.7) | |  |

**Table 2. The sleep pattern and school performance were affected by the COVID-19 home quarantine in narcolepsy patients (NT1、NT2）**

As shown in Table 2, NT1 patients delayed get up time (7:25 ± 1:21 vs. 6:28 ± 0:49, P < 0.001) and increased TST (7.64± 1.64 vs.6.83 ± 1.36, P < 0.001), increased nocturnal sleep quality (PSQI Score, 4.06 ± 2.37 vs. 5.41 ± 2.7, P = 0.004) during home quarantine. However, although it was not statistically significant when comparing the rankings of final exam scores between the two periods (42.7% ± 29.33% vs. 53.66% ± 26.73%, P = 0.001) , there was a percentage increase in the rankings during home quarantine. And a lower score of self-rated school/work affected by illness (SDS1 score, 2.47 ± 2.05vs. 3.76 ± 2.87, P = 0.015) were observed. Among NT2 patients, their academic performance was improved (41.1% ± 28.71% vs. 51.05% ± 28.49%, P = 0.029), but their sleep patterns did not change significantly.

| Characteristics | During the COVID-19 home quarantine | | After the COVID-19 home quarantine | | During the home quarantine VS.  After the home quarantine | | | |
| --- | --- | --- | --- | --- | --- | --- | --- | --- |
|  | NT1(n=34) | NT2 (n=12) | NT1 (n=34) | NT2 (n=12) | NT1 (n=34) | | NT2 (n=12) | |
|  | Mean ± SD | Mean ± SD | Mean ± SD | Mean ± SD | x^2^/t | P | x^2^/t | P |
| Weight gain, (%) | 17(50) | 5 (42) | 11 (32.3) | 4 (26.6) | 0.444 | 0.505 |  | 1 |
| Prolonged sleep  at night, (%) | 19 (55.8) | 5 (42) | NA | NA | NA | NA | NA | NA |
| Regular medicine, (%) | 9 (26.5) | 1 (8.3) | NA | NA | NA | NA | NA | NA |
| Percentile ranking | 42.7% ± 29.33%^a^ | 41.1% ± 28.71%^b^ | 53.66% ± 26.73%^c^ | 51.05% ± 28.49%^d^ | -1.934 | 0.065 | -2.543 | 0.029 |
| SDS1 score | 2.47 ± 2.05 | 2.75 ± 2.86 | 3.76 ± 2.87 | 3.67 ± 3.11 | -2.567 | 0.015 | -1.778 | 0.101 |
| Clinical symptoms | | | | | | | | |
| Sleepiness, (%) | 30 (88.2) | 11 (91.7) | 30 (88.2) | 10(83.3) |  | 1 |  | 1 |
| Emotional behavior, (%) | 34 (100) | 0 | 34 (100) | 0 |  | 1 |  | NA |
| Hallucination, (%) | 18 (52.9) | 0 | 14 (41.1) | 0 |  | 1 |  | NA |
| Sleep paralysis, (%) | 20 (58.8) | 4 (33.3) | 19 (55.8) | 2 (16.7) | 0.762 | 0.383 |  | 0.64 |
| Degree of sleepiness | | | | | | | | |
| Number of naps | 2.94 ± 1.3 | 2.92 ± 2.75 | 2.62 ± 1.18 | 3.08 ± 2.91 | -1.484 | 0.138 |  | 1* |
| Duration of each nap, min | 18.09 ± 9.54 | 21.67 ± 10.08 | 15.88 ± 10.32 | 15.83 ± 14.75 | -1.624 | 0.104 | -1.614 | 0.106* |
| ESS score | 13.53 ± 5.9 | 10.58 ± 4.21 | 13.85 ± 6.12 | 10.75 ± 5.41 | -0.281 | 0.781 | 0.118 | 0.908 |
| Emotional behaviors | | | | | | | | |
| Inability, (%) | 30 (88.3) | 0 | 31 (91.2) | 0 |  | 1 |  | 1 |
| Open mouth, (%) | 17 (50) | 0 | 17 (50) | 0 |  | 1 |  | 1 |
| Nod, (%) | 22 (64.8) | 0 | 21 (61.8) | 0 |  | 1 |  | 1 |
| Tumble, (%) | 12(32.4) | 0 | 12(32.4) | 0 |  | 1 |  | 1 |
| UNS score | 17.18 ± 6.21 | 9.5 ± 3.29 | 17.74 ± 7.03 | 9.67 ± 3.92 | -0.161 | 0.872* | -0.171 | 0.865* |
| Night sleep structure | | | | | | | | |
| Bed time, hh:mm | 22:41 ± 1:02 | 22:03 ± 0:46 | 22:35 ± 0:37 | 22:07 ± 0:46 | -0.823 | 0.410* | -0.862 | 0.389* |
| Sleep latency, min | 11.5 ± 10.74 | 10.67 ± 6.08 | 8.97 ± 6.34 | 13.33 ± 12.98 | -1.837 | 0.066* | -0.921 | 0.357* |
| Get up time, hh:mm | 7:25 ± 1:21 | 7:15 ± 1:05 | 6:23 ± 0:49 | 6:44 ± 0:5 | -3.676 | ＜0.001* | -1.845 | 0.065* |
| TST, h | 7.64 ± 1.64 | 8.42 ± 1.74 | 6.83 ± 1.36 | 7.7 ± 1.44 | 3.986 | ＜0.001 | -1.931 | 0.053* |
| PSQI score | 4.06 ± 2.37 | 3.33 ± 2.61 | 5.41 ± 2.7 | 4.08 ± 3.06 | -3.114 | 0.004 | -2.085 | 0.059 |
| GAD-7 score | 4.32 ± 4.4 | 3.42 ± 4.21 | 6 ± 4.66 | 4.5 ± 6.1 | -1.742 | 0.091 | -0.539 | 0.590* |
| PHQ-9 score | 7.97 ± 5.13 | 4.08 ± 4.29 | 8 ± 5.27 | 5.25 ± 4.58 | -0.024 | 0.981* | -1.556 | 0.146 |

*Wilcoxon signed-rank test, Paired-samples t test for ranks; x2: McNemar's Chi-squared test; t: Paired-samples t test. NT1, Narcolepsy type 1; NT2, Narcolepsy type 2; SDS1, Sheehan Disability Scale; ESS, Epworth Sleepiness Score; UNS, Ullanlinna Narcolepsy Scale; TST, Total sleep time; PSQI, Pittsburgh Sleep Quality Index; GAD-7, Generalized Anxiety Disorder-7; PHQ-9, Patient Health Questionnaire-9; n, number; SD, standard deviation.
